# GDM and Nutrition—Answered and Unanswered Questions—There’s More Work to Do!

**DOI:** 10.3390/nu11081940

**Published:** 2019-08-17

**Authors:** David Simmons

**Affiliations:** Macarthur Clinical School, Western Sydney University, Sydney, NSW 2560, Australia; da.simmons@westernsydney.edu.au

**Keywords:** gestational diabetes mellitus, pregnancy, lifestyle intervention, randomised controlled trial, healthy eating, physical activity, overweight, prevention

## Abstract

Gestational Diabetes Mellitus (GDM) is the commonest medical pregnancy complication, and a growing problem around the world as the obesity epidemic continues. Ways to prevent GDM are urgently required, the management of GDM still poses many unanswered questions, and the postpartum prevention of the progression of GDM to type 2 diabetes remains a challenge. With GDM, the impact of any intervention on the offspring is always a major concern. Nutritional interventions come to the fore as one of our few levers in reducing the short-term pregnancy risk and long-term cardiometabolic risks to both mother and child. This special issue reports on the relationship between several nutrients and foods and the development and management of GDM, behavioural strategies to enhance lifestyle choices, the issues raised by prior bariatric surgery and ways to screen for GDM. The work identifies further unanswered questions over nutritional strategies to reduce the impact of GDM.

The proportion of pregnancies complicated by gestational diabetes mellitus (GDM) has been increasing since at least the 1980s [1]. This burden has increased in parallel with the epidemic of type 2 diabetes, itself a consequence of the growing numbers of men and women who are overweight and obese [1]. Of course, the simple answer to excess adiposity, or excess calorie balance, is to reduce energy intake and/or increase energy expenditure to create a negative energy balance. In reality, in a modern world, within its obesogenic environment, lifestyles that limit non-work time and where opportunities for physical activity and affordable healthy eating can be limited, achieving this balance can be very difficult. Alongside the complex endocrine and behavioural mechanisms to manage energy balance [2], any inherited and/or acquired “processes” that promote increased insulin secretory capacity, or reduce insulin need, during pregnancy may have a chance of reducing the risk of GDM, if sufficient to overcome the large increase in insulin resistance that occurs [3]. Similarly, such “processes” may alter the need for medication in GDM, the birth outcomes, and long-term cardiometabolic risks for the mother and her offspring.

Preventing and managing GDM is substantially different to available approaches for managing weight and hyperglycaemia outside of pregnancy. Many medications are either harmful to the growing fetus, or their toxicity is uncertain, with substantial caution over their use [4]. Not only is weight loss not recommended due to its potential negative impact on fetal growth and development, there are clear guidelines on the amount of gestational weight gain (GWG) there should be by trimester and body mass index (BMI) at conception [5].

It is in this setting that managing nutrition comes to the fore as one of our few levers for preventing and managing GDM, the progression to type 2 diabetes, and, with these, potentially the life-long risk of cardiometabolic disease in the offspring. This special issue “Nutrition and Gestational Diabetes” comprises 14 peer-reviewed papers [6,7,8,9,10,11,12,13,14,15,16,17,18,19] that provide insight into different nutritional aspects relating to GDM. These papers address diet, gestational weight gain, and dietary patterns associated with the risk of GDM in the mother and/or offspring (*n* = 8), and with “better” GDM outcomes, including the long-term risk to the offspring (*n* = 2). The remaining papers review approaches to predict GDM (*n* = 1), screen for and manage GDM after bariatric surgery (*n* = 1), or support lifestyle change to prevent progression to type 2 diabetes after GDM (*n* = 1). One paper assesses the relationship between healthy eating and cardiometabolic risk in the offspring of mothers with GDM.

So, which diet is best? The St Carlos GDM Prevention Study [6] showed that among 874 Spanish women, a high adherence to a Mediterranean diet was associated with a 0.35 (95% CI 0.18–0.67) risk of developing GDM compared with women with low adherence. The earlier analysis of adding extra virgin olive oil and pistachios to the diet had previously found a 0.75 (0.57–0.98) risk [20]. The women had a mean baseline BMI of approximately 24 kg/m^2^, entered the trial at 8–12 weeks gestation, and had fewer large for gestational age (LGA) babies (adjusted odds ratio 0.19 (0.07–0.57)). Similarly, in the Chinese Prospective Birth Cohort Study [8] among 1014 women (median pre-pregnancy BMI <23 kg/m^2^), the “traditional eating pattern” (a high vegetable, fruit, and rice intake) was associated with a lower risk of GDM (0.40 (0.23–0.70) using food diary). A whole grain-seafood pattern was associated with a greater risk of GDM (1.73 (1.10–274)). Conversely, the metabolic profile (BMI, triglycerides, waist circumference) was poorer among the 9–16-year-old offspring of Danish women (*n* = 1234 mother-offspring dyads) with GDM (*n* = 608) who did not eat fish during pregnancy [8], suggesting that the seafood/fish associations may be markers for other behaviours/circumstances.

Meanwhile, the Norwegian Longitudinal Study [9] found among 702 women (40 with GDM) few dietary differences between women who developed GDM and those that did not. Similarly, Looman et al. from the Netherlands [10] found no consistent correlation between diet quality and glycaemia (as assessed by the Dutch Healthy Diet 2015 Index score). Weak correlations were found between fasting glucose and both diet quality and total iron intake (both *p* < 0.05). A significant caveat to some studies remains the use of food frequency questionnaires to assess dietary intake.

Which diets may make things worse? King and colleagues showed in a mouse model that a maternal low protein diet was associated with changes in the pancreatic islets of Langerhans, including altered glucagon and insulin expression and a low beta cell mass [11]. Farabi and Hernandez [12] meanwhile suggested that a lower carbohydrate diet (40% of total calories) among women with GDM, while reducing postprandial glucose, might increase fasting glucose, possibly through increased free fatty acids and worse insulin resistance. Interestingly, this paradoxical finding was supported by the DALI (Vitamin D And Lifestyle Intervention for GDM prevention) randomised controlled trial (RCT) [21], where European (across nine countries) overweight and obese women without GDM also developed a higher fasting glucose on a lower carbohydrate diet.

These papers on which food and nutritional constituents add to the current knowledge on lifestyle approaches to prevent GDM currently indicate that GDM is likely to only be preventable if commenced in (or before) the first trimester, or in some population subgroups [22]. Another premise is that the development of GDM is not related to the nutrients within the diet, but through excess calories overall, leading to excess GWG. The DALI post hoc analysis looks at this premise in more detail [13]. Across most lifestyle GDM prevention studies, mean GWG limitation (between controls and intervention groups) is up to 2 kg. In the post hoc analysis, selecting only those sites achieving a greater GWG limitation (>3 kg), even a mean GWG limitation of 4.3 kg by 35–37 weeks, was associated with no reduction in fasting, 1 h or 2 h glucose, or GDM by 24–28 weeks. However, there was a reduction in LGA (16.7% vs. 4.5%, usual care vs. healthy eating and physical activity). Another possible mechanism by which diet may increase the risk of GDM is through changes in the microbiome. Ponzo et al. provided a useful review of the current state of knowledge, suggesting that there remains discordant evidence over which changes in bacteria (type and richness) occur in pregnancies complicated by GDM [14]. Possible dietary interactions with the microbiome that may influence the risk of GDM have been suggested, along with a lower fibre intake, vegetarianism, and high fat/saturated fat intake. Unfortunately, recent trials of probiotics to change gut flora and reduce GDM have been unsuccessful [23].

Two behavioural approaches to changing diet are described in this special issue. The first looked at using antenatal motivational interviewing intervention to prevent GDM (DALI) [15], and the possible mediators of lifestyle change. Outcome expectancy, risk perception, task self efficacy, and social support were measured at baseline, 24–28 weeks and 35–37 weeks gestation. Task self efficacy and outcome expectancy were associated with reduced sugar drink consumption, while satisfaction with social support and outcome expectancy were associated with portion size. The other behavioural intervention used text messaging and activity monitors within a pilot RCT to reduce risk factors for postpartum progression to type 2 diabetes [16], a major problem that still seeks effective, scalable, and sustainable solutions. Carbohydrate consumption was reduced, and the text messaging was seen as useful by the women, but the activity monitors, although found to be useful, were not used consistently by many of the women. While these behavioural interventions clearly had an impact, they remained limited and more work is needed. An alternative approach for the obese is bariatric surgery. While still requiring changes in lifestyle, one way of supporting reduced energy intake is with bariatric surgery. In the review by Benhalima et al. [17], bariatric surgery was shown to reduce the risk of GDM, but the review emphasises that GDM can still occur and that micronutrient supplementation is needed before, during, and after pregnancy. Women who have had bariatric surgery may develop symptoms from dumping on the oral glucose tolerance test (OGTT), and are recommended to have instead a fasting glucose at the first visit and capillary glucose monitoring at 24–28 weeks gestation. The penultimate paper in the special issue also touched on the use of alternative screening tests [18] and showed poor utility of the fructosamine test to predict second trimester and postpartum glycaemia. The final paper [19] looked at 142 children with a mean age of 6–7 years and found that a healthy diet is associated with a better cardiometabolic health profile in children of women with previous GDM, including a lower risk of being overweight or obese.

Overall, we still seek better nutritional approaches to prevent and manage GDM and its postpartum progression to type 2 diabetes in the mother, and to protect the short- and long-term risk to the offspring. As a Guest Editor, I would like to thank all of the authors for contributing to this special edition, which will hopefully provide useful insights into what is known, and yet to be learnt, in the “hot topic” of nutrition and gestational diabetes. A special thanks goes to the Nutrients publishing team for all of their help.
